# Age and Grip Strength Predict Hand Dexterity in Adults

**DOI:** 10.1371/journal.pone.0117598

**Published:** 2015-02-17

**Authors:** Jason A. Martin, Jill Ramsay, Christopher Hughes, Derek M. Peters, Martin G. Edwards

**Affiliations:** 1 Functional Neuroimaging Group, Department of Radiology, University Hospital Bonn, Sigmund-Freud Str. 25, 53127, Bonn, Germany; 2 Division of Neuropsychology, Hertie-Institute for Clinical Brain Research, Centre for Integrative Neuroscience, Eberhard Karls University, Hoppe-Seyler-Str. 3, 72076, Tübingen, Germany; 3 School of Health and Population Sciences, University of Birmingham, Birmingham, United Kingdom; 4 Institute of Sport & Exercise Science, University of Worcester, Worcester, United Kingdom; 5 Faculty of Health & Sport Sciences, University of Agder, Kristiansand, Norway; 6 Psychological Sciences Research Institute, Université Catholique de Louvain, Louvain-la-Neuve, Belgium; 7 Institute of Neuroscience, Université Catholique de Louvain, Louvain-la-Neuve, Belgium; University of Rome Foro Italico, ITALY

## Abstract

In the scientific literature, there is much evidence of a relationship between age and dexterity, where increased age is related to slower, less nimble and less smooth, less coordinated and less controlled performances. While some suggest that the relationship is a direct consequence of reduced muscle strength associated to increased age, there is a lack of research that has systematically investigated the relationships between age, strength and hand dexterity. Therefore, the aim of this study was to examine the associations between age, grip strength and dexterity. 107 adults (range 18-93 years) completed a series of hand dexterity tasks (i.e. steadiness, line tracking, aiming, and tapping) and a test of maximal grip strength. We performed three phases of analyses. Firstly, we evaluated the simple relationships between pairs of variables; replicating the existing literature; and found significant relationships of increased age and reduced strength; increased age and reduced dexterity, and; reduced strength and reduced dexterity. Secondly, we used standard Multiple Regression (MR) models to determine which of the age and strength factors accounted for the greater variance in dexterity. The results showed that both age and strength made significant contributions to the data variance, but that age explained more of the variance in steadiness and line tracking dexterity, whereas strength explained more of the variance in aiming and tapping dexterity. In a third phase of analysis, we used MR analyses to show an interaction between age and strength on steadiness hand dexterity. Simple Slopes post-hoc analyses showed that the interaction was explained by the middle to older aged adults showing a relationship between reduced strength and reduced hand steadiness, whereas younger aged adults showed no relationship between strength and steadiness hand dexterity. The results are discussed in terms of how age and grip strength predict different types of hand dexterity in adults.

## Introduction

The hand is the most active and interactive part of the upper extremity. Hand dexterity is a term used to explain a range of different hand abilities and performances. These include reaction time; hand preference; wrist flexion speed; finger tapping speed; aiming; hand stability and arm stability (e.g., [1]). From these, four main factors are considered as the most characteristic and reliable for the evaluation of hand dexterity. These include: (i) steadiness; (ii) tracking; (iii) aiming (where typically the participant points to a target object), and (iv) tapping (where the participant taps as fast as possible for a set time period) ([1]; [2]; [3]; [4]; [5]).

The relationship between increased age and reduced hand dexterity has been widely reported in both the clinical and scientific literature. For example, [6] presented the first kinematic assessment that compared the reach-to-grasp movements for gender-matched groups of older aged (60–71 years, n = 12) and younger aged (18–25 years, n = 12) adults. Participants reached to grasp either a small cylinder using a precision grip or a large cylinder using a whole hand prehension. The actions performed by the two groups were similarly coordinated with similar times to peak for wrist velocity and acceleration from movement initiation (i.e. the transport component), and showed no differences in the size of the grip apertures used (i.e. object manipulation). However, the older aged participants made significantly slower movements than the younger aged adults, replicating previous findings (e.g., [7]; [8]; [9]; [10]; [11]). Although movement speed can be encapsulated within the term of hand dexterity, it is worth noting that slower movements with increased age may not necessarily correspond to a reduced performance for the other dexterity factors (e.g., aiming, stability etc.). This point will be investigated in the present study.

Despite numerous studies demonstrating a significant relationship between increased age and reduced hand dexterity, few studies have attempted to investigate the causes of the relationship. Instead, a common explanation is provided in the discussion of these papers stating that the relationship between increased age and reduced hand dexterity is likely caused by a decline in musculoskeletal strength and mass (see for example [12]; [13]; [14]; [15]; [16]; [17]; [18]). Much support can be found for these claims within related literature, with major reduction in muscle mass ranging from 20% to 45% in aging skeletal muscle (described as “sarcopenia of old age”; [19], p477) (see also [20]; [21]; [22]; [23]; [24]; [25]). More precise investigations of hand strength have also demonstrated diminished strength with increased age (e.g., [26]; [27]; [28]; [29]; [30]), with studies reporting that diminished hand strength appears associated to decreasing general muscle mass reduction ([13]; [21]; [25]; [31]). Furthermore, changes in muscle mass with increased age has been linked to changes in peripheral and central nerve conduction ([32]; [33]; [34]), proprioception ([35]) and changes in the human motor unit that relate to a degeneration of the nervous system ([23]; [36]); all of which are likely to impact hand dexterity.

Given the overwhelming evidence for relationships between age and hand dexterity, and between age and grip strength, it is perhaps surprising that there are very few studies that have investigated the relationships between age, grip strength and hand dexterity together in one study. Instead, the few studies considering the three factors in a single study have investigated differences in these factors for different age groups of participants. For example, Marmon et al. [17] tested three groups of adult participants (young 18–36, middle 40–60 and older ≥ 65 years aged adults) on measures of index finger abduction, precision pinch, and hand grip strength and measures of hand dexterity with the Grooved Pegboard test, the game Operation, a scissor task and a tracing task. The results showed significant differences between the young and older age groups for the measures of index finger abduction, precision pinch and handgrip strengths, and further for the measures of hand dexterity. A further analysis showed evidence of significant associations among similar tests (i.e. the strength measures; the steadiness measures and the four function measures) and between different measures. As a final multiple regression analysis showed that the time to complete two functional measures, the Grooved Pegboard and the game Operation were significantly predicted by index finger and grip strength or by pinch steadiness, index finger steadiness and grip strength respectively.

The aim of the current study was to extend these findings and investigate, using regression analyses, the relationships and interactions between age, grip strength and hand dexterity in adults. We hypothesised to replicate previous research by showing negative relations between age and strength, and age and hand dexterity, with increased age being related to reduced strength and hand dexterity. We also hypothesised a positive relationship between strength and dexterity (so that reduced strength was related to reduced hand dexterity). In a second phase of analyses, we then sought to extend the research findings by determining the variance of age and strength on the different components of dexterity (i.e. steadiness; tracking; aiming and tapping). In a final phase of analyses, we explored interactions in age and strength on hand dexterity.

## Methods

### Ethics Statement

All participants gave written informed consent to participate in the study, and the study was approved by the School of Sport and Exercise Sciences, University of Birmingham ethical committee in accordance with the ethical standards established by the 1964 Declaration of Helsinki.

### Participants

A total of 107 participants, aged 18–93 years were tested (60 female: mean 50 ± 21 years; range 18–86 years, and 57 male: mean 48 ± 18 years; range 20–93 years). All participants were recruited from the city of Birmingham (UK) following advertising the project at local community social groups and by giving short presentations about the project. Prior to giving an invitation to participate in the study, potential participants were pre-screened via an informal verbal discussion to determine eligibility. The inclusion criteria were that participants must be 18+ years of age, able to travel unaided to the University of Birmingham (i.e. by walking, driving or using public transport) and reported no muscular skeletal, arthritis or neurological problems. This information was collected using a purpose written self-report health questionnaire to assess for arthritis, and an informal verbal interview, all performed immediately prior to the testing sessions. These criteria were used to ensure that only participants in good ‘health’ were tested and that healthy age effects could be explored without being potentially confounded by ill health influencing the results. For clarity, good ‘health’ was classified as the absence of any diagnosed physical diseases, mental illnesses, recent surgeries or hospitalisation, feelings of illness, no reported sign of arthritis and with a high score (27/30) on the Mini Mental State Examination [37]. Any participants not meeting the criteria were not included in the study.

### Apparatus and Design

For the main study, we assessed hand dominance (Edinburgh handedness inventory: [38]) and measured grip strength for both hands and dexterity for the dominant hand only. To measure grip strength we used a handgrip dynamometer (Takei Scientific Instruments, Japan) that has been shown to be an easy, fast and reliable method ([39]). Participants were positioned sitting upright, with their elbows by their left and right sides, and flexed to right angles. A neutral wrist position held the dynamometer in their hand to be tested. The handle of the dynamometer was adjusted so that the base of the dynamometer was positioned with the rest on first metacarpal (heel of palm), and the handle rested on the middle of the four fingers prior to testing. The results were recorded as kilograms taken from the digital display of the dynamometer to the nearest 0.1 kg. The digital display of the dynamometer displayed the maximum strength within a trial and the value was reset to zero before each subsequent measurement.

**Fig 1:**
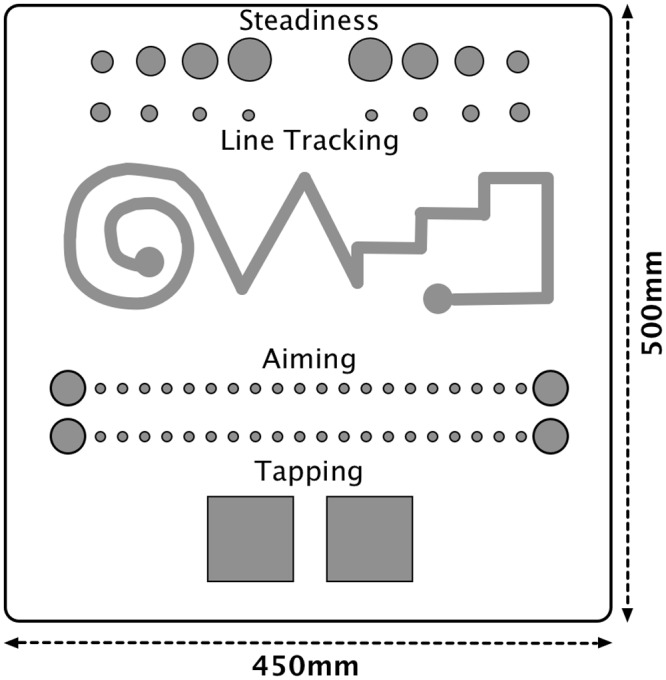
A schematic diagram of the motor performance series work panel.

Dexterity was measured using the ‘Vienna Test System: Motor Performance Series Workboard’ (VTS: MLS; Lafayette Instrument, Model 64030, Lafayette, IN [40]; developed by Schoppe [41]; and based on Fleishman’s factor-analytic examinations of fine motor abilities in arms: [42]; See Fig. 1). The test is a standardised diagnostic tool used to provide valid and reliable assessments of hand dexterity (see [43]; [44]; [45]). The VTS part of the test consists of a main computer with test interface management software and the MLS part consists of a peripheral panel containing a variety of different tests. Test-retest reliability has been reported for the MLS to range from. 60 to. 94, dependent on the dexterity measure used ([45]). From the MLS tests, we used measures of steadiness, line tracking, aiming and tapping (explained in the procedure section); thus measuring the different components most characteristic for the evaluation of hand dexterity performance ([1]; [2]; [3]; [4]; [5]). The MLS was placed on a wooden testing table, and participants sat on a stable wooden chair with back support in a normal sitting posture with slightly bent forearms. Participants were positioned at the mid-point of the table, perpendicular to the MLS and throughout the tests were instructed not to make contact with the wooden table or MLS (i.e. so that no additional postural support was achieved).

### Procedure

Participants took part in one session that lasted approximately ninety minutes. At the beginning of the session, after providing informed consent, participants were given the set of questionnaires that were used for the inclusion criteria. If a participant was not compatible with the inclusion criteria, they were not included in the study. Following the questionnaires, and confirmation of their inclusion, participants performed the grip dynamometry assessment. The participant squeezed the dynamometer with maximum effort for at least 5 seconds. Handgrip strength was tested three times with a minimum of 60 seconds rest was provided for recovery between each attempt. Maximum grip strength was recorded as the highest value of three trials. Note that the maximum value was used rather than the mean of three trials allowing for a measure that was independent of fatigue, which may have been possible in some of the participants.

Next participants completed the series of fine motor tests using the VTS: MLS. Before commencement of the selected MLS tests, each test was verbally explained and demonstrated to the participant with a brief period provided for the participant to familiarise themselves with each test. The VTS management software controlled the order of the MLS tests and recorded all measurements. The same order of tests was used for all participants (as the software was unable to randomise the order of the tests). At the end of each test sequence, a break was provided.

In the fixed sequence of tests, the first MLS test was the steadiness test. This involved the participant to vertically insert a pen stylus (2mm diameter) into a pre-assigned hole (5.8mm diameter) and then hold the stylus as still as possible for 32 seconds without touching the sides or bottom of the hole. If the participant touched the sides they simply had to correct and position the stylus in the centre of the hole and continue until the end of the 32 seconds period. Measurements recorded by the software were the number of times the stylus came into contact with the sides or the bottom of the hole and the total duration of the contact time. The second test was line tracking. This involved the insertion of the pen stylus into a grooved track and tracing the pen stylus through the track without touching the sides or the bottom. The test was undertaken at a self-selected pace. If the sides were touched, participants had to correct the position of the pen stylus and continue along the grooved track until the end. Measurements recorded were the number of times the stylus touched the side or bottom, the total duration of any touches and the total duration of the tracking. The third test was the aiming test. This involved the participant touching 20 brass disks (5mm diameter) that were arranged in a row with the pen stylus. The distance between each disk was 4mm. The participant was instructed to tap one circle in the row after the other using the pen stylus, moving from right to the left across the disks. Measurements recorded were the number of correct taps, the number of incorrect taps, the total duration of any incorrect tap and the total duration to complete. The final task was tapping. This involved the participant tapping a square plate (40 mm^2^) with the pen stylus as many times as within 32 seconds. The measurement recorded was the total number of taps. The VTS software recorded all measurements. Each dependent variable consisted of a composite performance score calculated using the Fleishman-factor of weighted variables from each measure (e.g., a composite of the number of touch errors and the total duration of any touches for line tracking etc.) (See [3]; [45]; [46]).

### Data Analyses

The data analysis was undertaken in three phases. In the first phase, we report the simple relationships between two of the variables using simple regression analysis; between age and strength; age and each dependent measure of the MLS; and strength and each dependent measure of the MLS. The aims of these first analyses were to replicate the existing findings in the literature. In the second phase of analysis, the three variables were compared together using standard multiple regression. In a third phase, we modified the variables to allow analyses of interactions. The independent variables of age and grip strength were mean centred to provide numerical stability (see recommendations by Cohen and Cohen [47]; and Aitken and West [48]). Prior to the standard Multiple Regressions (MR) preliminary analyses were conducted to ensure no violation of the assumptions of normality; linearity; multicollinearity and homoscedasticity occurred, using a criterion of p < 0.001 for Mahalanobis distance. No outliers among the cases were found. Then a standard MR was used to analyse the predictive variance between the independent variables of age and strength, and each dependent dexterity measure. Where both independent variables had a significant contribution to the dependent variable, a further standard MR analysis was undertaken using the mean centred data and the mean centred cross product of age and strength to test for an interaction (see [47]). In addition, post hoc Simple Slopes Technique analyses were used to further investigate the interaction (see [47]; [48]). This was done to determine specific values of (centred) age, that were one standard deviation below the sample mean, the mean, and one standard deviation above the mean; in this instance to represent low aged, mean aged and high aged adults, respectively, and the same to represent low strength, mean strength and high strength adults, respectively. These values were then entered into the regression equation Y = (b_1_+b_3_Z)X + (b_2_Z+b_0_) to calculate the data to plot the simple slopes for analysis, where X = Strength and Z = Age. All statistical analyses were performed using SPSS v21 (SPSS, Chicago, Illinois, USA), except the Simple Slopes Technique that was performed in Matlab (Mathworks, Massachusetts, USA) using a bespoke script.

## Results

### Phase 1: Simple Linear Regressions

**Fig 2:**
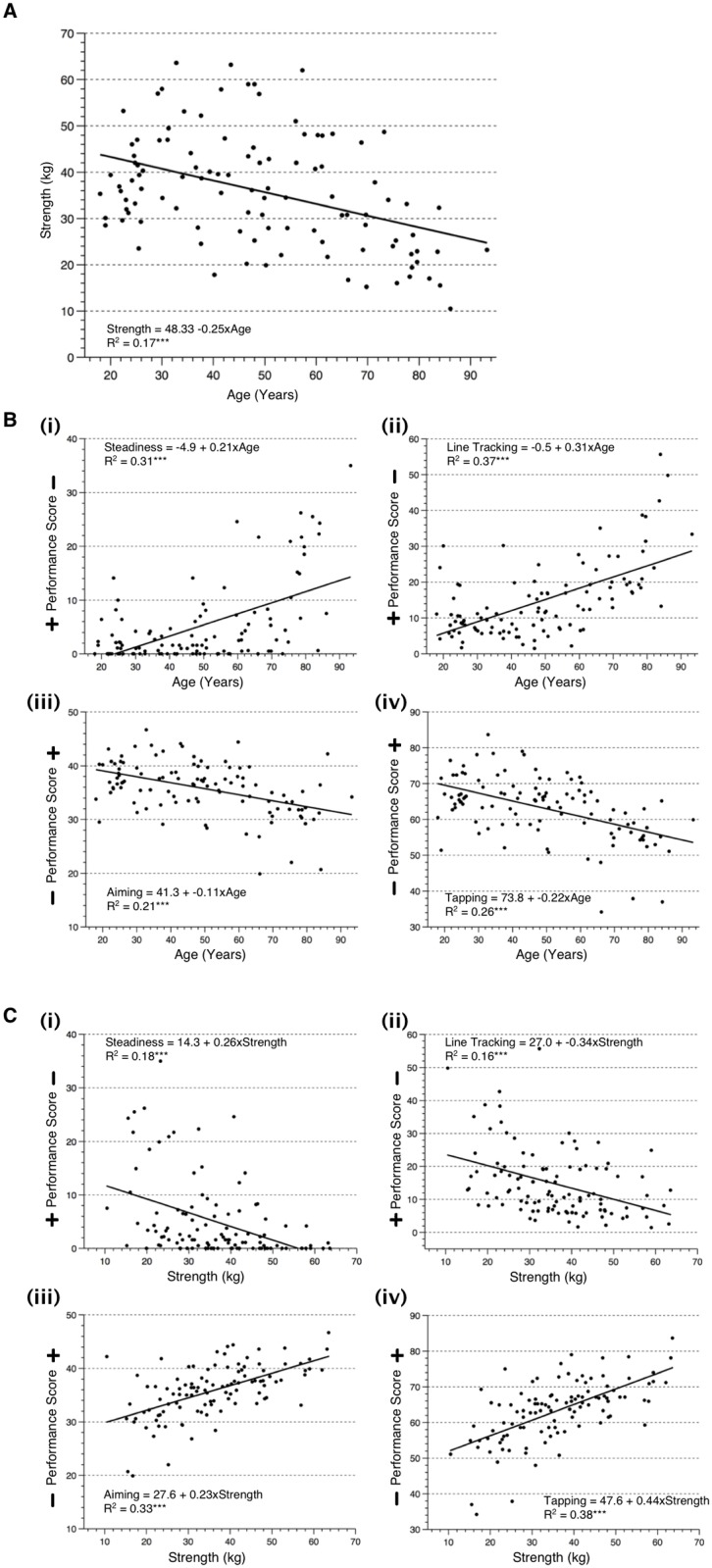
Nine scatterplots to illustrate the relationships between: (A) age and grip strength; (B) the dependent measures of hand dexterity and age; and (C) the dependent measures of hand dexterity and strength. Also shown on each plot is the best-fit simple regression line and the linear regression equation (***p<0.001, n = 107). NB: Dependent measures are represented in the following order: (i) steadiness; (ii) tracking; (iii) aiming; (iv) tapping; with the direction of a positive/negative performance score indicated.

Analyses of the simple relationships between variables showed a significant negative relationship between age and grip strength (r = -0.42, p<0.001; see Fig. 2a; see also S1 Fig.). As hypothesised, this showed that increased age was related to decreased strength, with the linear best fit showing a decline of 0.25 kg for each year of age. There were also significant simple relationships between age and each dependent measure of the MLS (see Fig. 2b) (steadiness: r = 0.56, p<0.001; line tracking: r = 0.61, p<0.001; aiming: r = -0.46, p<0.001, and: tapping: r = 0.51, p<0.001) and between strength and each dependent measure of the MLS (see Fig. 2c) (steadiness: r = -0.42, p<0.001; line tracking: r = -0.40, p<0.001; aiming: r = 0.57, p<0.001, and: tapping: r = 0.62, p<0.001).

### Phase 2: Multiple Regression (MR) Analysis

**Fig 3:**
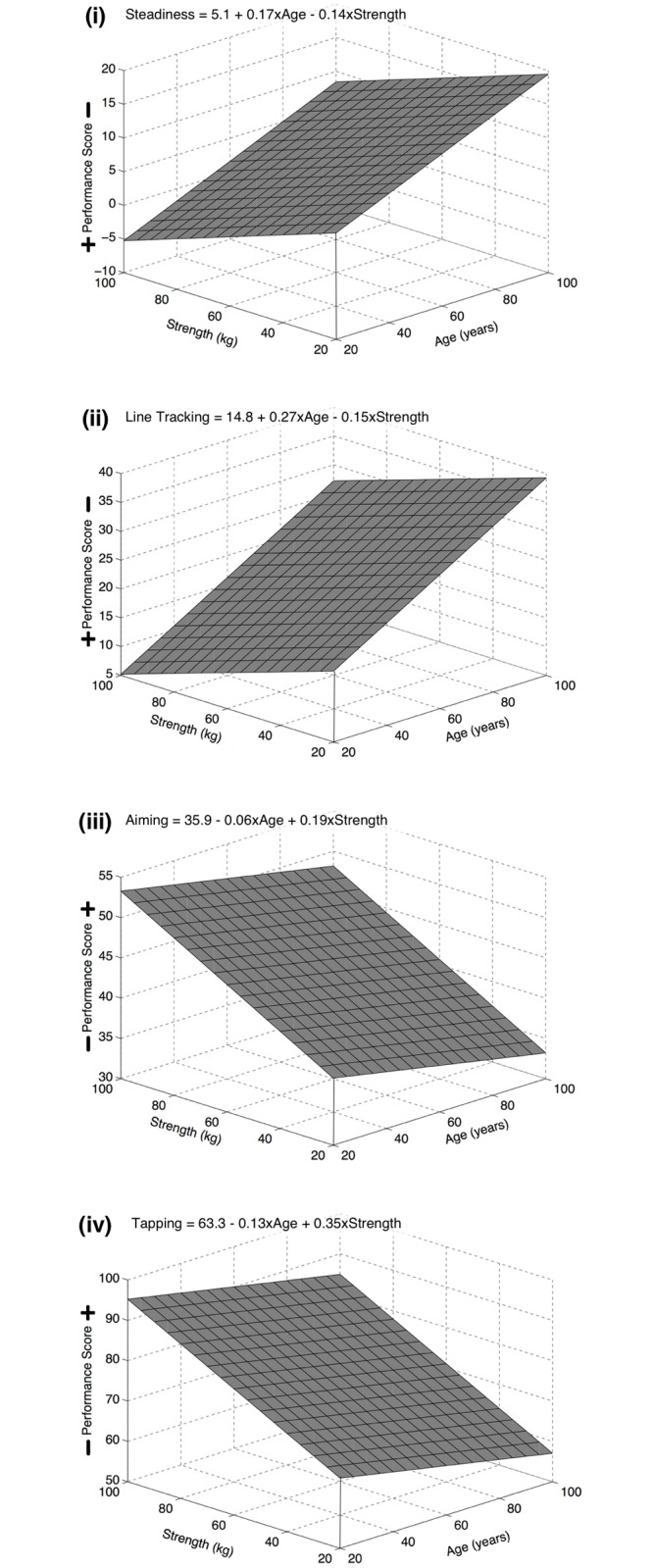
Three-dimensional plot of the best-fit multiple regression plane relating age and strength for each dependent measures of hand dexterity (i: steadiness; ii: line tracking; iii: aiming; iv: tapping).

The results of the MR analyses are presented in Tables 1 and 2. Table 1 shows the results of the standard MR analyses. This showed that both variables of age and strength significantly predicted the variance of each hand dexterity dependent variable. However, the results showed that age predicted more variance than strength for the hand dexterity measures of steadiness and line tracking, whereas strength predicted more variance than age for the hand dexterity measures of aiming and tapping. Table 2 shows the results of the MR analyses in which the product of the age and strength variables only showed a significant interactions effect for age and strength on the hand dexterity dependent variable of steadiness. Fig. 3 presents these interaction effects between age and strength for each of the hand dexterity dependent variables in four separate MR plane fit plots (the data points are not plotted).

**Table 1:** The results of the multiple regression analysis for age and strength on each movement dexterity task.

Variables	Parameter B	SE	Standardised β	Model R^2^	sr^2^ (unique)
Steadiness					
Intercept	5.12	0.58			
Age	0.17 ***	0.03	.46		0.18
Strength	- 0.14 **	0.05	-.23	0.35 ***	0.04
Line Tracking					
Intercept	14.80	0.78			
Age	0.28 ***	0.04	.53		0.23
Strength	- 0.15 **	0.7	-.18	0.40 ***	0.03
Aiming					
Intercept	35.86	0.37			
Age	- 0.06 ***	0.02	-.26		0.06
Strength	0.19 ***	0.03	.46	0.39 ***	0.18
Tapping					
Intercept	63.28	0.62			
Age	- 0.13 ***	0.03	-.30		0.08
Strength	0.35 ***	0.06	.49	0.46 ***	0.20

* p < 0.05;

**p < 0.01;

***p < 0.001.

**Table 2:** The results of the multiple regression interaction analyses (age, strength and age x strength) for each dexterity task.

Independent Variables	Parameter B	SE	Standardised β	Model R^2^	sr^2^ (unique)
Steadiness					
Intercept	4.39	0.63			
Age	0.14 ***	0.03	.38		0.10
Strength	-0.12 *	0.05	-.20		0.03
Age X Strength	-0.01 *	0.00	-.22	0.35 ***	0.04
Line Tracking					
Intercept	14.80	0.87			
Age	0.25 ***	0.05	.49		0.18
Strength	-0.14 *	0.07	-.17		0.02
Age X Strength	-0.01	0.00	-.11	0.40 ***	0.00
Aiming					
Intercept	35.86	0.42			
Age	-0.07 **	0.02	-.27		0.05
Strength	0.19 ***	0.03	.47		0.18
Age X Strength	0.00	0.00	-.03	0.39 ***	0.00
Tapping					
Intercept	63.25	0.69			
Age	-0.12 **	0.04	-.28		0.06
Strength	0.34 ***	0.06	.49		0.19
Age X Strength	0.00	0.00	.06	0.46 ***	0.00

* p < 0.05;

**p < 0.01;

***p < 0.001.

### Phase 3: Simple Slopes Post-Hoc Analyses

**Fig 4:**
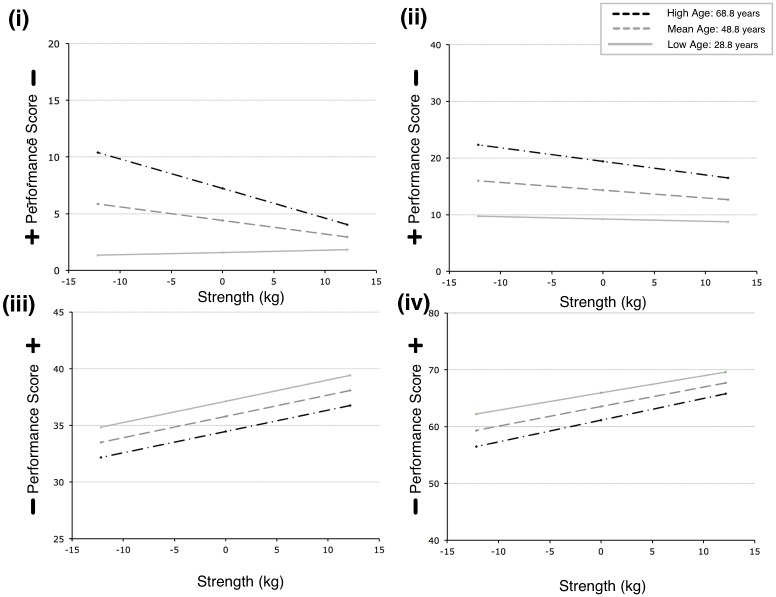
Four plots illustrating the simple slope post hoc interaction analyses: (i) strength and steadiness; (ii) strength and line tracking; (iii) strength and aiming; (iv) strength and tapping. Data plotted are mean centred age ± 1 SD, where minus one standard deviation represents younger adults (Low age) and plus one standard deviation represents older adults (High age). Note that the direction of positive performance score is indicated.

The interaction effects were analysed using Simple Slopes post-hoc analyses. For the interaction effect for steadiness dexterity, the high aged (68.8 years) and mean aged (48.8 years) adults showed a significant positive relationship between strength and steadiness, whereas lower aged (28.8 years) adults showed no relation between strength and steadiness. No other interaction analyses were performed (see Fig. 4).

## Discussion

The goal of the present study was to examine the relation between age, grip strength and hand dexterity. The motor tests were chosen to reflect those factors most characteristic and reliable for the evaluation of hand dexterity. Here, we firstly replicated previous literature showing significant simple relationships between age and strength, age and hand dexterity, and strength and hand dexterity. Increased age was significantly associated with decreased strength and decreased hand dexterity, and decreased strength was significantly associated with decreased hand dexterity. MR analyses showed that performance on steadiness and line tracking dexterity tests were better explained by age than strength, but aiming and tapping dexterity were better explained by strength than age. Analyses of the interaction between age and strength for hand dexterity showed a significant interaction for steadiness hand dexterity. Post-hoc analyses of the steadiness interaction highlight that the older and mean aged adults showed reduced hand steadiness with reduced strength, but that there was no effect for younger adults.

The finding of a simple relationship between age and grip strength replicated previous studies in the literature (e.g., [25]; [26]; [28]; [29]; [30]; [49]; [50]; [51]; [52]; [53]; [54]). Furthermore, the finding that grip strength reduced by 0.25 kg or 1.4% per year was roughly consistent to previous findings showing that grip strength declines annually between 1%–1.5% ([26]; [30]). We know that grip strength is linked to reductions in muscle mass ([13]; [21]; [25]), and that age associated reductions in muscle mass are also consistently linked to changes in the properties of muscle activation and muscle recruitment that likely have an impact on hand dexterity. For example, notable age-related changes in muscle properties are: a slowed rate of muscle contraction ([55]; [56]); a slowed neural conduction velocity ([57]; [58]); and increased muscle antagonist coactivation that’s necessary to stabilise a joint during a movement ([13]; [59]), but has the by-product of also restricting joint movement. The simple relations between age and the hand dexterity measures of steadiness, line-tracking, aiming and tapping also replicated existing literature, showing a reduced performance with increased age ([17]; [50]; [52]; [60]; [61]; [62]). For example, Marmon et al. [17], recently evaluated finger dexterity using the Grooved Pegboard test along with measures of strength, steadiness and other hand functions. For all of their measures, they observed that increased age was associated with reduced performance and that progressive declines in strength (i.e. grip, pinch and index finger abduction) are observed with increased age. In the final simple relation that we investigated, we showed that all hand dexterity tasks significantly declined with decreased strength. This indicates that adults with weaker grip typically had worse movement dexterity than stronger adults.

While our study is not the first study to show associations between age and hand dexterity or strength and hand dexterity (as discussed above), this is the first study that provides evidence of associations between the three factors of age, strength and hand dexterity using standard MR analyses. These analyses showed that age and strength significantly moderated hand dexterity, with the two variables explaining between 35% and 46% of the different hand dexterity tasks variance. This finding is consistent with the findings from the simple two variable correlation analyses. As adults increased in age, their strength and hand dexterity declined. Interestingly, these results showed two main findings. This was that hand dexterity performance on steadiness and line tracking were better explained by age than strength, but aiming and tapping dexterity were better explained by strength than age. We discuss each of these findings in turn.

The hand dexterity tasks of steadiness and line tracking both rely on a stable control of the arm and hand, and eye-hand coordination with visual guidance. Strength had little predictive value to the variance of steadiness and line tracking hand dexterity, suggesting that other factors may have influenced the result. The other factors could have included vision, or cognitive capacity for online visual guidance; though we excluded participants with impaired visual acuity, vision or cognitive capacity; it is clear that all these factors deteriorate with age ([63]). Also, Marmon et al. [17], who also reported reduced steadiness hand dexterity with older compared to younger aged adults, found the effect irrespective of whether the task was performed with or without visual feedback, suggesting that factors of reduced visual guidance with increased age may be less relevant. An alternative explanation for the poor steadiness and line tracking hand dexterity with increased age could be a consequence of the control of hand muscle force, known to fluctuate more in older adults ([17]; [64]; [65]; [66]; [67]; [68]). These moderations in muscle control are thought to involve changes in average motor unit force output ([65]; [69]), antagonist coactivation ([69]; [70]; [71]), and motor unit discharge variability ([69]; [72]), and are unrelated to muscle strength *per se*. A similar explanation could be that tremor-related movements caused by irregular fluctuations in muscle and limb displacement ([73]; [74]; [75]) reduced steadiness and line tracking hand dexterity performance. We propose that future studies should investigate the role of muscle control and tremor on steadiness and line tracking hand dexterity in ageing.

The results of the MR analyses showed that the variance of aiming and tapping hand dexterity tasks was more explained by grip strength than age. This suggests that dexterous actions that rely on the fast and precise coordinated movement control of the hand, wrist, elbow and shoulder, such as that of aiming and tapping, appear directly associated to hand grip strength. Although this is the first time that this point is made, it is well known that the successful coordination of muscles during movement execution depends on well-regulated muscular forces, sensory information and body scheme ([76]; [77]; [78]). The presence of fluctuations in muscle force during a movement influences the capacity to produce the intended movement correctly, causing compensations within the movement. In aiming and tapping, the thumb and index finger must pinch the stylus object with a minimum force that must be greater than the friction force required to prevent object slipping when lifting the object ([79]; [80]). The performance of the swift aiming and tapping movements then requires large (and sequenced) muscle activations with rapid accelerations of movement to respond to the task. At the same time, the thumb and index finger pinch of the stylus must coordinate force with the movement accelerations and the impact with the target. Therefore, reduced strength will lead to increases in the variability of force capability, causing increases in variability to the movement trajectory and the accuracy of the final position ([14]; [81]; [82]; [83]; [84]). These effects will be compounded in repeat performances of a task (e.g., the tapping task) in that fluctuations in muscle force during a voluntary contraction will cause an increase in the variance of movement kinematics from trial to trial ([14]; [85]; [86]; [87]; [88]; [89]; [90])

The suggestion in our data here is that having a higher level of grip strength will lead to a higher aiming and tapping ability. Currently, this view is speculative, but we propose that increased grip strength will be associated with fewer fluctuations in muscle force during movement execution, allowing for faster, more fluent, less variable and more stable successful successive movements. Support for our speculation is present in existing literature where increases in muscle strength, after just a few weeks of strength training, have been associated with decreases in force fluctuations during isometric and anisometric contractions in a number of muscles (e.g. first dorsal interosseus muscle and knee extensor muscles) ([91]; [92]; [93]; [94]) (for a review of mechanisms that contribute to differences in motor performance between young and old adults, see [14]). Additionally, individuals who regularly practice rapid, coordinated, goal directed movements, such as expert musicians, appear not to display the same age related decrements of dexterity that are typical of age matched non-musical adults ([95]; [96]). Such an observation suggests that regular exercise is possibly sufficient to maintain rapid, coordinated, goal directed dexterous actions (such as tapping and aiming). If correct, strength exercises in adults may be a simple preventative measure against hand dexterity decline, independent of age. We propose that future research should test where moderations in strength cause changes in rapid, coordinated, goal directed movements such as tapping and aiming hand dexterity.

A final novel finding of this paper was that age and strength variables interacted with the hand dexterity measure of steadiness. The post hoc analyses indicated that the mean aged and older aged adults showed a relationship where reduced strength was associated to reduced steadiness hand dexterity, but the younger aged adults showed no relationship between strength and steadiness hand dexterity. This interaction result was interesting, as it appeared to suggest that steadiness hand dexterity was moderated by strength, only for older adults. This finding needs further investigation. It is unclear whether strength training interventions would be beneficial to steadiness hand dexterity in the same way as the exercises proposed to improve aiming and tapping hand dexterity, discussed above. It may be that other factors such as muscle control may co-vary with grip strength, explaining the apparent strength effect here. Beyond strength, changes in touch sensation may be considered as a plausible factor to explain such changes; however, in light of a recent research study [50] it would appear that any age-related reductions in sensation and motor function cannot easily be inferred from each other, and as such do not offer a simple explanation.

Our study presents some novel findings and some factors that we propose future studies should include and consider. The finding that steadiness and line tracking hand dexterity was moderated more by age than strength suggests that another factor must contribute to the moderation in performance. In the discussion above, we suggested that muscle control and / or tremor may have been a causal factor, and we advocate the measure of these factors in future studies. Additionally, future studies should consider the effects of impaired visual acuity upon aspects of hand dexterity performance such as steadiness; here we had no in-depth assessment of visual acuity but simply controlled vision with a self-report of corrected vision and an informal discussion.

In this study, we also showed that aiming and tapping hand dexterity was moderated more by strength than age, and we proposed that future studies should investigate the effect of exercise on these types of action performance. Furthermore, our study could have included control for levels of physical activity or specific dexterity skills (i.e. musicians, jugglers, etc…), and we propose that future studies manipulate or control these factors. In this study, we only assess handgrip strength but have no measure of wrist, elbow or shoulder strength or power; arms movements during the experimental measures were free to reflect natural unsupported functional performance as such handgrip strength may not allow us to fully account for different task strategies of whole arm function that may be interesting for future studies. A final proposal for future studies is the investigation of hand dominance or differences in participant gender in these effects. We know that the dominant hand is usually stronger than the non-dominant hand, and also that males are stronger than females. However, it is currently unclear how these factors interact with age. We might expect that strength differences between the dominant and non-dominant hand show parallel effects in the age and hand dexterity relationship. However, it is unclear whether the same parallel effect will be present for males compared to females.

In conclusion, the present research showed that age and grip strength were all statistically significant predictors of hand dexterity, and that aiming and tapping hand dexterity (involving rapid, coordinated, goal directed actions) appeared to be moderated by the factor of grip strength more than age. This suggests that physical activity may improve particular types of hand dexterity. The research also showed that steadiness and line tracking hand dexterity performance was better explained by age than strength, and an interaction between age and strength on steadiness hand dexterity showed that mean to older adults were particularly influenced by strength. These latter findings need further investigation, with aim to determine what other factors than strength appear to moderate steadiness and line tracking hand dexterity. Future research into hand function should be complemented by other neurophysiological recordings that can provide insight into the mechanisms responsible for age-related differences in muscular control.

## Supporting Information

S1 Fig Scatterplot of age and grip strength for the non-dominant hand. (TIFF)
