# Supplementary figures and images for: Age and Grip Strength Predict Hand Dexterity in Adults
Source: PLoS One. 2015 Feb 17;10(2):e0117598. doi: 10.1371/journal.pone.0117598 (PMC4331509; doi:10.1371/journal.pone.0117598)

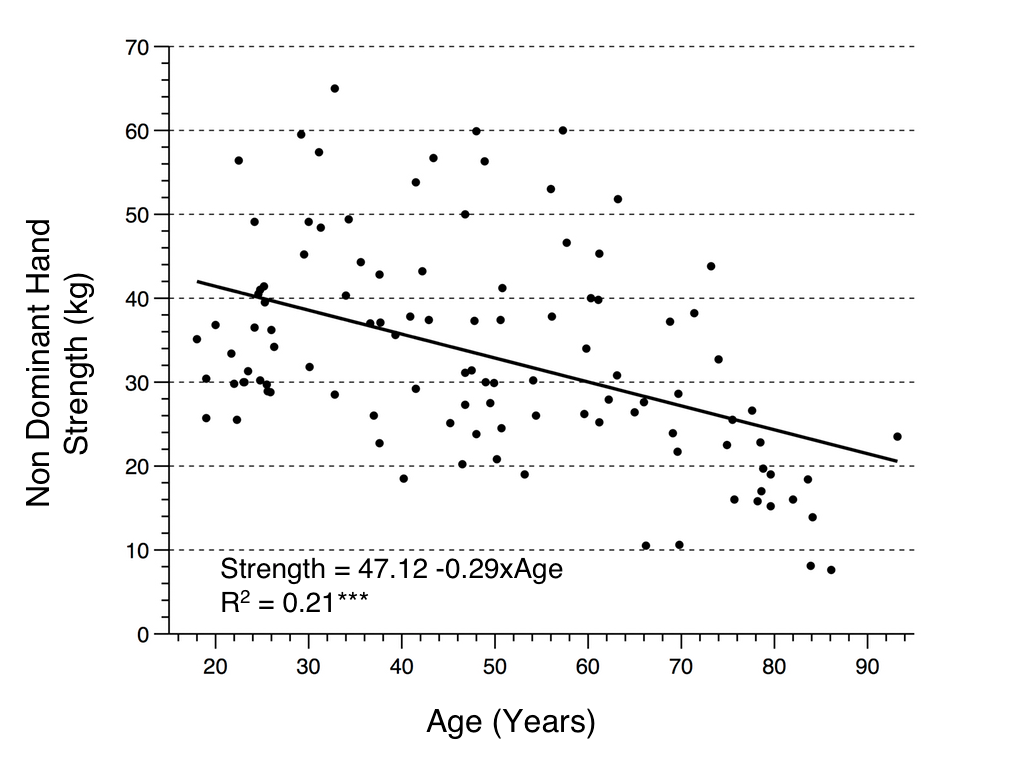

Supplement: S1 Fig — (TIFF) [file pone.0117598.s001.tiff]
